# Integrating Network Toxicology and Untargeted Metabolomics to Identify Candidate Mechanisms and Metabolic Markers of α-Amanitin-Induced Hepatorenal Injury in Mice

**DOI:** 10.3390/metabo16080551

**Published:** 2026-08-04

**Authors:** Haiyan Cui, Yue Guo, Wang Xin, Dan Shao, Meng Hu, Tao Wang, Dan Zhang, Zhiwen Wei, Keming Yun

**Affiliations:** 1 School of Forensic Medicine, Shanxi Medical University, Jinzhong 030600, China; 2 Shanxi Key Laboratory of Forensic Medicine, Jinzhong 030600, China; 3 Key Laboratory of Forensic Toxicology of Ministry of Public Security, Jinzhong 030600, China

**Keywords:** α-amanitin, network toxicology, metabolomics, hepatorenal injury, molecular docking

## Abstract

**Highlights:**

**What are the main findings?**

- Metabolomic pathway analysis of plasma from α-amanitin-poisoned mice preliminarily reveals that plasma metabolites follow a temporally dynamic progression across seven different groups.
- PTGS2 emerges as a potential candidate target; propionylcarnitine and 2-arachidonoylglycerol (2-AG) are the key associated metabolites.

**What are the implications of the main findings?**

- Similar to the clinical symptomatology, α-amanitin-induced liver and kidney injury follows a temporally dynamic evolutionary process, which may inform reference criteria for poisoning treatment.
- By integrating untargeted metabolomics with network toxicology, this study couples the phenotypic endpoints represented by differential metabolites with the regulatory networks mediated by potential candidate targets. This approach enables a multi-level elucidation of the mechanisms underlying α-amanitin-induced hepatorenal injury, which might provide a reference basis for early toxicity warning and precision intervention.

**Abstract:**

**Background/Objectives**: Mushroom poisoning is a leading cause of death from foodborne illness, and α-amanitin is its most potent toxin. However, the mechanisms and biomarkers of α-amanitin-induced hepatorenal injury remain inadequately understood. **Methods**: First, the potential candidate targets and metabolic pathways were identified using network toxicology. Subsequently, untargeted metabolomics was employed to screen for potential metabolic markers of poisoning in α-amanitin-treated mice across eight time groups: 0 h, 3 h, 8 h, 12 h, 24 h, 2 d, 4 d, and 7 d. Following this, a compound–reaction–enzyme–gene network was constructed based on the differential metabolites to identify relevant genes, which were integrated with the core candidate target genes derived from network toxicology. The findings were further validated by molecular docking and immunohistochemistry. **Results**: Network toxicology identified eight candidate targets associated with α-amanitin-induced combined hepatic and renal injury. Metabolomic analysis revealed that α-amanitin-induced hepatorenal injury followed a temporally dynamic progression, and that propionylcarnitine was the common differential metabolite across all time points. Integration of the network toxicology and untargeted metabolomics results indicated that PTGS2 served as a potential candidate target and 2-arachidonoylglycerol (2-AG) was selected as a potential differential metabolite in the combined hepatic and renal injury caused by α-amanitin. **Conclusions**: By integrating network toxicology and untargeted metabolomics, this study preliminarily reveals that α-amanitin-induced hepatorenal injury follows a temporally dynamic progression, which is consistent with the toxicological injury–response–adaptation model. PTGS2 is tentatively annotated as the potential candidate target, while propionylcarnitine and 2-AG are the potential candidate metabolites. These findings may provide valuable guidance for poisoning treatment and identification in cases of mushroom poisoning caused by *Amanita* species.

## 1. Introduction

Mushrooms are nutrient-dense fungi that are rich in protein, vitamins and dietary fiber and are highly valued in human diets for their immunomodulatory and antioxidant properties [1,2]. However, the close morphological resemblance between many edible and toxic species often leads to accidental ingestion, resulting in widespread cases of poisoning and fatalities worldwide [3,4,5]. Within this context, food poisoning cases attributed to the genus *Amanita* account for over 90% of all mushroom poisonings and exhibit a high fatality rate, posing a serious threat to public health [6,7].

The primary toxic components of *Amanita* species are amatoxins [8]. These toxins are rapidly cleared from the body and are typically difficult to detect in the blood 48 h after ingestion and generally undetectable in urine after 72 h, with only rare cases showing residual levels [9,10]. Among the amatoxins, α-amanitin is the most potent, with a lethal dose of 0.1 mg/kg in adults [11]. It is resistant to drying, high temperatures and extreme pH values, making it impossible to inactivate through ordinary cooking methods. Thus, poisoning incidents can still occur even when the mushrooms are thoroughly cooked [9,12]. The liver and kidneys are the primary target organs for α-amanitin [13,14]. The clinical progression typically follows a distinct pattern: an incubation period, an acute gastroenteritis period, a pseudo-recovery period and a fulminant hepatic failure period [15,16]. Currently, therapeutic options such as penicillin G and silibinin exhibit limited efficacy, and no specific antidotes are available. Consequently, the mortality rate among poisoned patients remains high [17].

At present, the toxic mechanisms of α-amanitin remain incompletely understood. The prevailing view is based on the classical theory, which posits that α-amanitin inhibits eukaryotic RNA polymerase II, thereby blocking mRNA synthesis and triggering cellular apoptosis [18]. However, studies have demonstrated that preventing the binding of α-amanitin to RNA polymerase II does not entirely alleviate toxin-induced tissue damage [19]. This discrepancy suggests that additional pathways may be involved in the pathogenesis of α-amanitin. Therefore, the mechanism of α-amanitin-induced hepatorenal injury requires further systematic investigation.

Network toxicology is an extension of network pharmacology [20]. It constructs compound–target–adverse effect interaction networks to analyze how exogenous chemicals perturb the topological structure of biological networks and trigger subsequent alterations in signaling pathways. This approach systematically elucidates the molecular mechanisms, toxic effects and potential risks of toxic substances [21,22]. However, network toxicology primarily relies on database mining and algorithmic predictions, which means that its results require further validation. Because this approach relies on static topological structures to predict toxicological effects, their ability to explain both time-dependent toxicological processes and dose-response relationships is limited [23].

Metabolomics serves as a direct reflection of an organism’s ultimate functional state at a specific time under defined physiological conditions. It offers high sensitivity, with a comprehensive perspective for capturing early, subtle toxicological responses and drug-induced mechanisms [24]. In recent years, the applications of metabolomics to identify potential poisoning biomarkers, which serve as evidence of antemortem toxin exposure, have become a research hotspot in forensic toxicology [25,26,27]. However, metabolomics only reflects changes in metabolite abundance in an organism under specific environmental conditions and at particular times, which complicates the direct elucidation of the causal relationship between the molecular mechanisms of toxins and their toxicity.

Given the existing limitations, this study employed network toxicology to investigate the targets and metabolic pathways associated with α-amanitin-induced hepatorenal injury. By integrating network toxicology and untargeted metabolomics, we systematically elucidated the multi-target and multi-pathway network mechanisms of α-amanitin-induced hepatorenal injury across three distinct levels: molecular interactions, signaling perturbations and metabolic phenotypes. Furthermore, we identified metabolic markers for α-amanitin exposure. This approach combined the mechanistic predictive power of network toxicology with the phenotypic validation offered by metabolomics, which was further substantiated through molecular docking and immunohistochemistry. Ultimately, this study aims to investigate the potential mechanisms of α-amanitin-induced hepatorenal injury from the molecular to the systemic level, providing a robust reference for poisoning treatment and identification in mushroom poisoning events caused by *Amanita* species.

## 2. Materials and Methods

### 2.1. Chemicals and Reagents

All reagents, including formic acid (Aladdin, Shanghai, China), methanol (Sigma-Aldrich, Buchs, Switzerland), and acetonitrile (Sigma-Aldrich, St. Louis, MO, USA), were of HPLC grade. α-amanitin (purity: 92.9%) was purchased from Tianjin Alta Technology Co., Ltd. (Tianjin, China), and 2-chloro-L-phenylalanine was obtained from Sichuan Weikeqi Biotechnology Co., Ltd. (Chengdu, China). For immunohistochemical analysis, the primary antibody was rabbit anti-mouse PTGS2 monoclonal antibody (supplier: HuaAn; lot number: ET1610-23; Hangzhou, China), used at a dilution of 1:1000; this antibody has been validated by the manufacturer for mouse tissue. The secondary antibody was goat anti-rabbit IgG-HRP (supplier: Abcam; lot number: ab6721; Shanghai, China), used at a dilution of 1:500. The sodium-heparin-coated tubes were purchased from Liuyang Sanli Medical Technology Development Co., Ltd. (Liuyang, Hunan, China).

### 2.2. Animals

Male Kunming (KM) mice, six weeks of age, weighing 30 ± 5 g, were obtained from the Laboratory Animal Center of Shanxi Medical University (SYXK晋2024-0007), Jinzhong, China. The total number of animals used was 144 KM mice. KM mice were the first application in this study and were randomly assigned to groups for free feeding and water intake under a 12 h light–dark cycle at a room temperature of 22 ± 2 °C and a relative humidity of 50–60%. After one week of acclimatization, they were randomly divided into groups for drug administration.

Based on the preliminary experimental results, we imported the metabolite concentration matrix into MetaboAnalyst for sample size simulation. Under the FDR < 0.05 criterion, we plotted a curve of number of significant metabolites vs. sample size and observed a rapid increase in the number of detected metabolites as the sample size increased from 6 to 8 per group. At *n* = 9, the curve clearly entered a plateau phase (with an additional increase in metabolite detection rate of <5%), which indicated that further increases in sample size yield minimal gains in identifying novel differential metabolites. Accordingly, we defined turning point 9 as the minimum required number of biological replicates per group.

A randomized grouping design was employed in this study. All mice meeting the inclusion criteria were randomly assigned to one of eight time-point groups (0 h, 3 h, 8 h, 12 h, 24 h, 2 d, 4 d, and 7 d) after completing acclimatization feeding and prior to drug administration, with nine mice per group. A random number was generated for each mouse using the RAND function in Microsoft Excel; the mice were then assigned to the respective groups in ascending order of the generated random numbers. The treatment groups received injections of α-amanitin at a dose of 0.2 mg/kg, while the control group received an injection of equal volume of physiological saline.

A one-way ANOVA was performed on the body weights of mice in each group after grouping, confirming no significant differences in initial body weight between groups (*p* > 0.05), thereby excluding the potential influence of body weight on the metabolome.

Upon arrival at the barrier facility, mice underwent a one-week period of adaptive housing during which their mental status, activity levels, food and water intake, and coat luster were monitored daily. Upon completion of the adaptive housing phase, all mice exhibited no abnormal clinical signs and demonstrated good health status.

To minimize the systematic impact of the cage effect on metabolomic results, after grouping, mice from the same time-point group were distributed across different cages (4–5 per cage, from different groups) to avoid systematic bias in the metabolic profile due to the cage effect. Each cage simultaneously housed individuals from different time-point groups, with cage positions on the housing rack arranged completely randomly to ensure balanced comparability among treatment groups in terms of microenvironmental conditions such as space, lighting and ventilation.

All animal experiments complied with the ARRIVE guidelines; the experimental procedures were approved by the Institutional Animal Care (SYDL2025014) and Use Committee of Shanxi Medical University and were conducted in strict accordance with the relevant animal welfare regulations in China.

### 2.3. Histopathological Test

The liver and kidney tissues were fixed in 4% paraformaldehyde, dehydrated through a graded series of ethanol (50%, 75%, 80%, 90%, 95%, and 100%), cleared in xylene, and embedded in paraffin. The resulting tissues were sectioned, stained with hematoxylin and eosin (H&E), and mounted with neutral balsam. Finally, the sections were examined under an Olympus BX40 microscope, and photomicrographs were taken using an Olympus DP71 digital microscope camera to assess histological damage to the liver and kidneys.

### 2.4. Network Toxicology Analysis

#### 2.4.1. Target Prediction

The 2D structure of α-amanitin was retrieved from the PubChem (https://pubchem.ncbi.nlm.nih.gov/) database and uploaded to the PharmMapper database for target gene prediction (accessed on 27 May 2025, https://www.lilab-ecust.cn/pharmmapper/). For PharmMapper, we retained the top 1000 mapped targets based on a Normalized Fit Score (NFSS) > 0.8 and only considered targets with a z-score > 2.0 relative to the mean fit of all hits. To identify potential toxicity targets, the keywords liver injury, hepatotoxicity, kidney injury and renal toxicity were used to query the GeneCards (accessed on 3 June 2025, https://www.genecards.org/) and Online Mendelian Inheritance in Man (OMIM, https://www.omim.org/) databases. For GeneCards, we applied a relevance score ≥ 10 (default median value) to filter potential targets. Genes with scores below this threshold were excluded to reduce false positives. The resulting datasets for hepatorenal injury were merged and deduplicated. Subsequently, the data were cross-referenced with the UniProt (accessed on 29 May 2025, https://www.uniprot.org/) database to map the identified proteins to their corresponding standardized gene nomenclature. All gene symbols were mapped to UniProt IDs using the UniProtKB Retrieve/ID mapping tool (accessed on 29 May 2025, https://www.uniprot.org/uploadlists/) with default settings (organism: Homo sapiens).

#### 2.4.2. Functional Enrichment and Pathway Analysis

The potential targets of α-amanitin were intersected with the identified liver- and kidney-injury-related targets to isolate the specific genes responsible for α-amanitin-induced hepatorenal injury. These candidate genes were uploaded to the STRING (accessed on 10 June 2025, https://cn.string-db.org/) database to construct a protein–protein interaction (PPI) network. PPI networks were retrieved with a combined confidence score threshold of 0.700; the network was restricted to homo sapiens with the default STRING network type.

This network was subsequently imported into Cytoscape software (version 3.9.1, Cytoscape Consortium, San Diego, CA, USA), where CentiScape analysis was employed to identify core target genes [28]. Hub nodes were identified by calculating degree, betweenness and closeness centralities using Cytoscape’s NetworkAnalyzer. Nodes with values exceeding the median + 1.5 × interquartile range (IQR) for all three metrics simultaneously were initially flagged as candidate hubs. Hub nodes were defined as those with degree ≥ 8.216216216216216, betweenness ≥ 41.29729729729731, and closeness ≥ 0.013464716294050655, with the intersection of high-scoring nodes across multiple centrality metrics prioritized as core targets. Nodes with degree = 0 (no interactions meeting the threshold) were excluded from the network to avoid isolated nodes that had no contributions to pathways analysis. Finally, these potential candidate targets were submitted to the Metascape database for Gene Ontology (GO) and Kyoto Encyclopedia of Genes and Genomes (KEGG) pathway enrichment analyses to elucidate the molecular mechanisms of α-amanitin-induced hepatorenal injury. The background gene set for GO and KEGG enrichment was defined as the entire human genome (protein-coding genes) annotated in the respective database, rather than the filtered gene list only. All enrichment *p*-values were adjusted using the Benjamini–Hochberg (BH) false discovery rate (FDR) method. An adjusted value of *p* < 0.05 was considered statistically significant.

### 2.5. Untargeted Metabolomics Analysis

#### 2.5.1. Animal Experiments

After administration, the mice were randomly divided into eight time-point groups: 0 h, 3 h, 8 h, 12 h, 24 h, 2 d, 4 d, and 7 d (*n* = 9 for each group). For each time point, the experimental groups were paired and compared exclusively with the saline group collected at the exact same corresponding time point; the total number of animals used was 144 KM mice. Based on preliminary experimental results, mice in all treatment groups received an intraperitoneal injection of α-amanitin at a dose of 0.2 mg/kg (equivalent to 0.186 mg of active α-amanitin). The α-amanitin solution was prepared at a fixed concentration, and the administration volume for each mouse was individually calculated based on its body weight to deliver the target dose. Mice in the saline control group received an equal volume of sterile saline, adjusted according to the same body-weight criteria. The animals at different time groups were independent in the experimental design. For each group, blood samples were collected from the medial canthus venous plexus into sodium heparin anticoagulant tubes for subsequent metabolomics and biochemical tests. In addition, the liver and kidneys were collected and weighed to calculate organ indices and to prepare for subsequent histopathologic sections.

#### 2.5.2. Organ Indices

During the breeding periods, the fur color, activity status, body weights and other relevant conditions of the mice were recorded for each group (0 h, 3 h, 8 h, 12 h, 24 h, 2 d, 4 d, and 7 d). After euthanasia, the liver and kidneys were collected and weighed to calculate the organ indices.

Organ index = (organ weight/body weight) × 100

#### 2.5.3. Biochemical Index Assay

Blood samples were collected in anticoagulant tubes and centrifuged at 4 °C for 15 min at 1006× *g*. The supernatant was aliquoted and stored at −80 °C. Prior to analysis, the thawed samples were centrifuged again, and biochemical tests of liver and kidney function were conducted using corresponding assay kits. Liver function markers included alanine aminotransferase (ALT), aspartate aminotransferase (AST), total bilirubin (TBIL), and direct bilirubin (DBIL) levels. The kidney function markers included blood urea nitrogen (BUN) and creatinine (CREA).

#### 2.5.4. Histopathological Analysis

All the original sections were independently and blindly rescored by two investigators. The final score for each indicator was determined by the average of the two investigators’ scores. Tissue damage was scored using a semiquantitative scoring system. For the liver sections, assessment indicators included hepatocellular necrosis, vacuolar degeneration, inflammatory cell infiltration and congestion. For the kidney sections, assessment indicators such as tubular necrosis, casts, interstitial inflammation, and glomerulosclerosis were included. Each indicator was scored on a 0–4 scale (0 = no lesion, 1 = mild/focal, 2 = moderate/multifocal, 3 = severe/diffuse, 4 = very severe). Five non-overlapping high-power fields (×400) were randomly selected from each slice along the long axis of the tissue for observation, the average of the scores of each field was used as the final score of the slice.

#### 2.5.5. Sample Preparation

A 100 μL aliquot of each plasma sample was slowly thawed at 4 °C then mixed with 20 μL of a 0.1 mg/mL internal standard solution of 2-chloro-L-phenylalanine by vortexing. Subsequently, 300 μL of a pre-chilled methanol and acetonitrile mixture solution (*v*/*v* = 2:1) was added to precipitate the proteins. After vortexing for 1 min, the mixture was subjected to low-temperature ultrasonication for 15 min, incubated at −20 °C for 30 min, and centrifuged at 18,900× *g* for 10 min at 4 °C. The resulting supernatant was filtered through a 0.22 μm organic membrane prior to the instrumental analysis. To monitor instrument stability and data reliability during the experiment, quality control (QC) samples were prepared by pooling 20 μL from each plasma sample.

#### 2.5.6. Untargeted Metabolomics Analysis Conditions

Metabolite separation and detection were performed using an UltiMate™ 3000 system (Thermo Fisher Scientific, Waltham, MA, USA). Chromatographic separation was achieved on an ACQUITY UPLC HSS T3 column (2.1 mm × 150 mm, 1.8 µm, Waters, Milford, MA, USA) at 25 °C. The mobile phase consisted of solvent A (0.1% formic acid in water) and solvent B (0.1% formic acid in acetonitrile). The gradient elution was programmed as follows: 2% solvent B was held constant from 0 to 1.5 min, followed by a linear increase to 50% B between 1.5 and 10 min, then increased to 98% B from 10 to 17.5 min. The 98% B composition was maintained from 17.5 to 19.5 min, after which it was reduced back to 2% B between 19.5 and 20.5 min; finally, 2% B was held from 20.5 to 22 min. The flow rate was 0.3 mL/min, and the injection volume was 5 μL.

Mass spectrometry was conducted using an electrospray ionization source in both positive and negative ion modes. Source parameters were as follows: sheath gas flow rate at 30 Arb, auxiliary gas flow rate at 10 Arb, auxiliary gas heater temperature at 350 °C, capillary temperature at 325 °C and spray voltage at 3.8 kV. Data acquisition for MS^1^ and MS^2^ spectra was performed using the Full Scan-ddMS^2^ mode. The MS^1^ scan range was *m*/*z* 60–1000 Da, and the MS^2^ scan range was *m*/*z* 25–1000 Da. The collision energies were set at 20, 40, and 70 in the normalized collision energy (NCE) mode.

#### 2.5.7. Data Processing and Multivariate Analysis

Raw data were processed using the Thermo Compound Discoverer software (version 3.1, Thermo Fisher Scientific, USA) to obtain the compound molecular formulas, ion modes, retention times, *m*/*z* values and peak areas. MS^1^ and ddMS^2^ spectra were matched against the mzCloud database, and the compounds with the highest matching scores were selected for initial identification. The data were imported into SIMCA software (version 14.1.0, Sartorius Stedim Data Analytics AB, Umeå, Sweden) for multivariate analysis. Unsupervised principal component analysis (PCA) was performed to assess sample clustering and dispersion; the clustering of quality control (QC) samples was used to evaluate data reliability. Orthogonal partial least-squares discriminant analysis (OPLS-DA) was applied to enhance separation between groups, with 200 permutation tests to prevent overfitting and a permutation test to validate the model. Differential metabolites were based on the following criteria: variable importance in projection (VIP) > 1 from the OPLS-DA model, *p*-value < 0.05 from *t*-tests, and fold change (FC) ≥ 1.5 or ≤0.67.

#### 2.5.8. Metabolites Identification

The identification of metabolites was performed by matching the results against the Human Metabolome Database (HMDB, accessed on 15 December 2025, http://www.hmdb.ca/), PubChem and the Kyoto Encyclopedia of Genes and Genomes (KEGG, accessed on 15 December 2025, https://www.genome.jp/kegg/) to determine the compounds’ names. Differential metabolites identified at each time point were imported into the MetaboAnalyst database (version 6.0, accessed on 15 December 2025, https://www.metaboanalyst.ca/) for metabolic pathway analysis; pathways with impact > 0 were selected for further interpretation.

### 2.6. Integrated Analysis of Metabolomics and Network Toxicology

#### 2.6.1. Compound–Reaction–Enzyme–Gene Network

Differential metabolites identified at each time point were imported into Cytoscape software, and the Metscape plugin was used to construct a compound–reaction–enzyme–gene network [29]. In this network, genes associated with the differential metabolites were identified and compared with the potential candidate targets of α-amanitin-induced hepatorenal injury previously enriched via network toxicological analysis, with the aim of pinpointing overlapping candidates.

#### 2.6.2. Molecular Docking

The 2D structure of α-amanitin obtained from PubChem was imported into Chem3D (version 20.0, PerkinElmer Informatics, Waltham, MA, USA) to generate its 3D structure. The overlapping target proteins were retrieved from the Protein Data Bank (PDB, https://www.rcsb.org/) database to obtain their corresponding protein structures. Molecular docking between α-amanitin and these protein structures was performed using CB-Dock2 (accessed on 15 December 2025, http://clab.labshare.cn:10380/cb-dock2/php/blinddock.php); the docking results were visualized from a 2D perspective using the Discovery Studio Visualizer (version 4.5, BIOVIA, Dassault Systèmes, San Diego, CA, USA).

#### 2.6.3. Immunohistochemical Validation

A total of three mice per group were used; three non-serial sections were subsequently collected from each animal. The liver and kidney tissue specimens were fixed in formalin, embedded in paraffin, and sectioned at 4 μm. Following deparaffinization and rehydration, antigen retrieval was performed using a citrate buffer under microwave heating. Endogenous peroxidase activity was quenched with 3% H_2_O_2_, followed by blocking with 5% bovine serum albumin. The sections were incubated with primary antibodies overnight at 4 °C and subsequently incubated with secondary antibodies at room temperature.

Immunoreactivity was visualized using diaminobenzidine substrate; the sections were counterstained with hematoxylin, mounted and observed under a light microscopy (NIKON ECLIPSE). Negative controls were prepared by substituting the primary antibody with phosphate-buffered saline. Five non-overlapping fields of view were randomly captured per section at 400× magnification for analysis. Images were analyzed using Image-Pro Plus software (version 6.0, Media Cybernetics, Rockville, MD, USA). The fields were selected using a randomization strategy, avoiding tissue edges, folds and damaged areas. Imaging parameters were set as follows: exposure time 50 ms, gain 1.5× and objective numerical aperture 0.60. All parameters were kept identical across all captured fields to ensure uniform acquisition conditions.

The protein expression levels were quantified as the average optical density (AOD) of the positively stained regions. AOD was calculated as: AOD = integrated optical density of positive areas/area of positive regions.

### 2.7. Statistical Analysis

Statistical analysis and data visualization were performed using GraphPad Prism (version 9.0.0, GraphPad Software, San Diego, CA, USA). All data were presented as the mean ± standard deviation (SD) from at least three independent experiments. Differences among three or more groups were assessed by one-way ANOVA, while comparisons between two groups were performed using Student’s *t*-test. Differences with a *p*-value < 0.05 were considered significant, and those with a *p*-value < 0.01 were considered highly significant. Considering the distinct physiological characteristics, metabolomic data were normalized to time-paired controls to minimize circadian interference, while biochemical and weight data were normalized to the initial baseline (0 h) to reflect cumulative changes.

## 3. Results

### 3.1. Histopathological Sections of Liver and Kidney Injury Induced by α-Amanitin

Preliminary histological examination revealed tissue edema and scattered inflammatory cell infiltration in the liver. Renal histopathology showed pronounced inflammatory cell infiltration, dilation of Bowman’s capsule, thinning of the renal tubular walls, and enlargement of the tubular lumina. Furthermore, homogeneous eosinophilic staining, nuclear loss, and cellular lysis were evident within the renal tubules.

### 3.2. Network Toxicology Analysis

#### 3.2.1. Targets of α-Amanitin-Induced Liver and Kidney Injury

**Figure 1:**
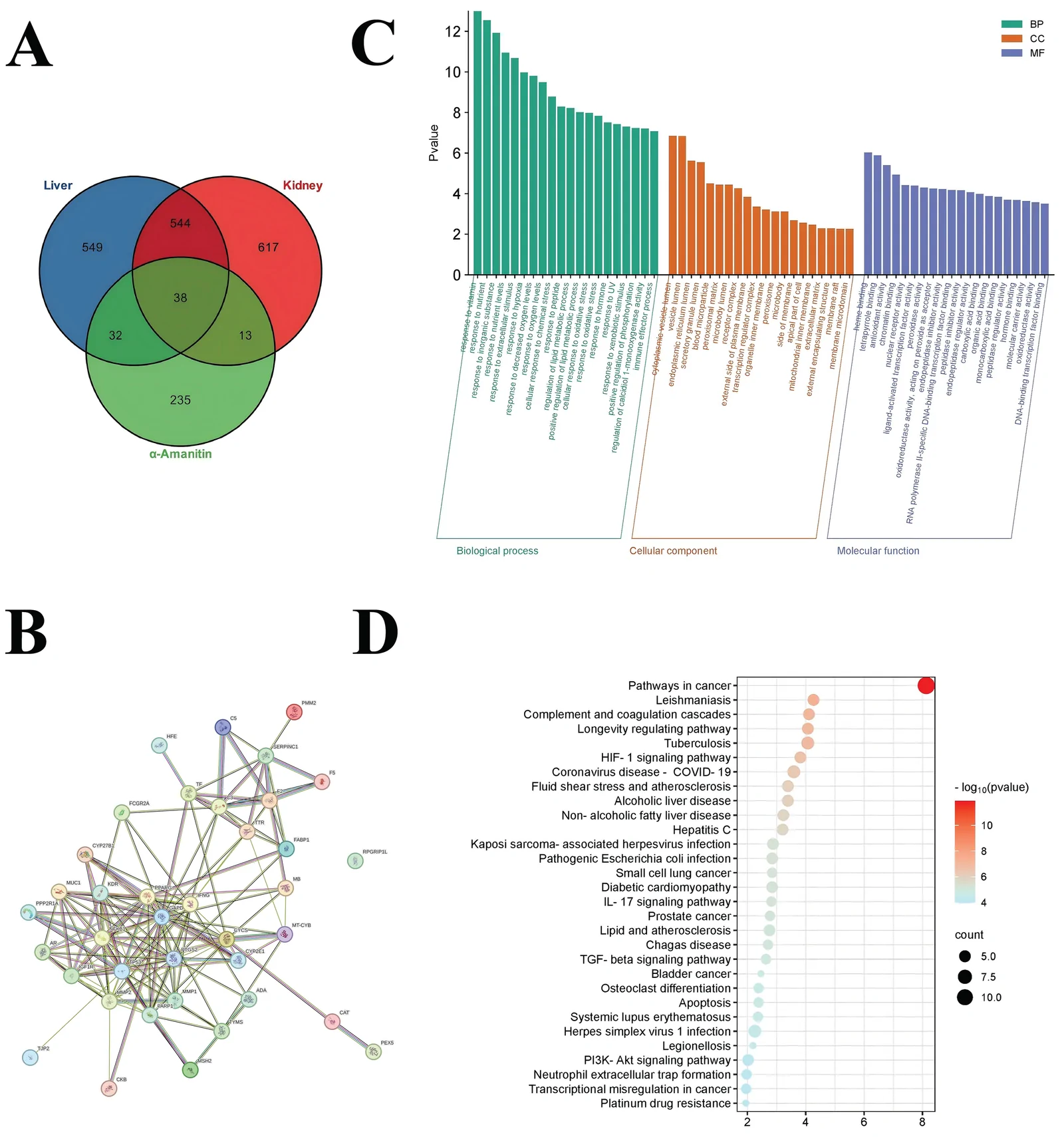
Network toxicology. (**A**) Venn diagram of target genes for liver and kidney injury induced by α-amanitin. (**B**) PPI network diagram of liver and kidney damage caused by α-amanitin. (**C**) GO enrichment analysis diagram of α-amanitin-induced liver and kidney injury. (**D**) KEGG enrichment analysis diagram of α-amanitin-induced liver and kidney injury.

The 2D structure of α-amanitin was retrieved from the PubChem database and uploaded to PharmMapper, yielding 1000 structurally predicted proteins. After mapping these to standardized gene nomenclature via the UniProt database, 318 structure-predicted targets for α-amanitin were selected. Simultaneously, liver and kidney toxicity-related target genes were retrieved from the GeneCards and OMIM databases. After deduplication, the toxicity targets of liver and kidney were obtained; the numbers were 1163 and 1212, respectively (Figure 1A). By intersecting the structure-predicted targets of α-amanitin with these organ-specific toxicity targets, a total of 70 hepatic and 51 renal targets were obtained as being specifically associated with α-amanitin-induced hepatorenal injury. These overlapping targets were imported into the STRING platform to construct PPI networks (Figure 1B), which were subsequently analyzed in Cytoscape using CentiScape to identify core target genes. This analysis yielded seventeen potential candidate targets for liver toxicity and twelve potential candidate targets for kidney toxicity, with an overlap of eight potential candidate targets between the two organs.

#### 3.2.2. Pathways Involved in α-Amanitin-Induced Liver and Kidney Injury

Liver and kidney injury induced by α-amanitin involved a total of 329 biological processes (BPs), with the most prominent including response to hypoxia and response to oxidative stress. A total of 18 cellular components (CCs) were implicated, mainly including vesicle lumen, endoplasmic reticulum lumen, peroxisomal matrix, receptor complex and transcription regulator complex. For molecular functions (MFs), 42 terms were identified, including heme binding, transcription co-regulator binding, peroxidase activity, transcription coactivator binding and oxidoreductase activity. In addition, 63 KEGG pathways were enriched, primarily involving complement and coagulation cascades, longevity-regulating pathways, the hypoxia-inducible factor-1 (HIF-1) signaling pathway, non-alcoholic fatty liver disease and the IL-17 signaling pathway. The top 20 pathways with *p* < 0.05 were selected to generate a KEGG pathway bubble diagram (Figure 1C,D).

### 3.3. Untargeted Metabolomics Analysis

#### 3.3.1. Liver and Kidney Organ Indices at Different Time Points

**Figure 2:**
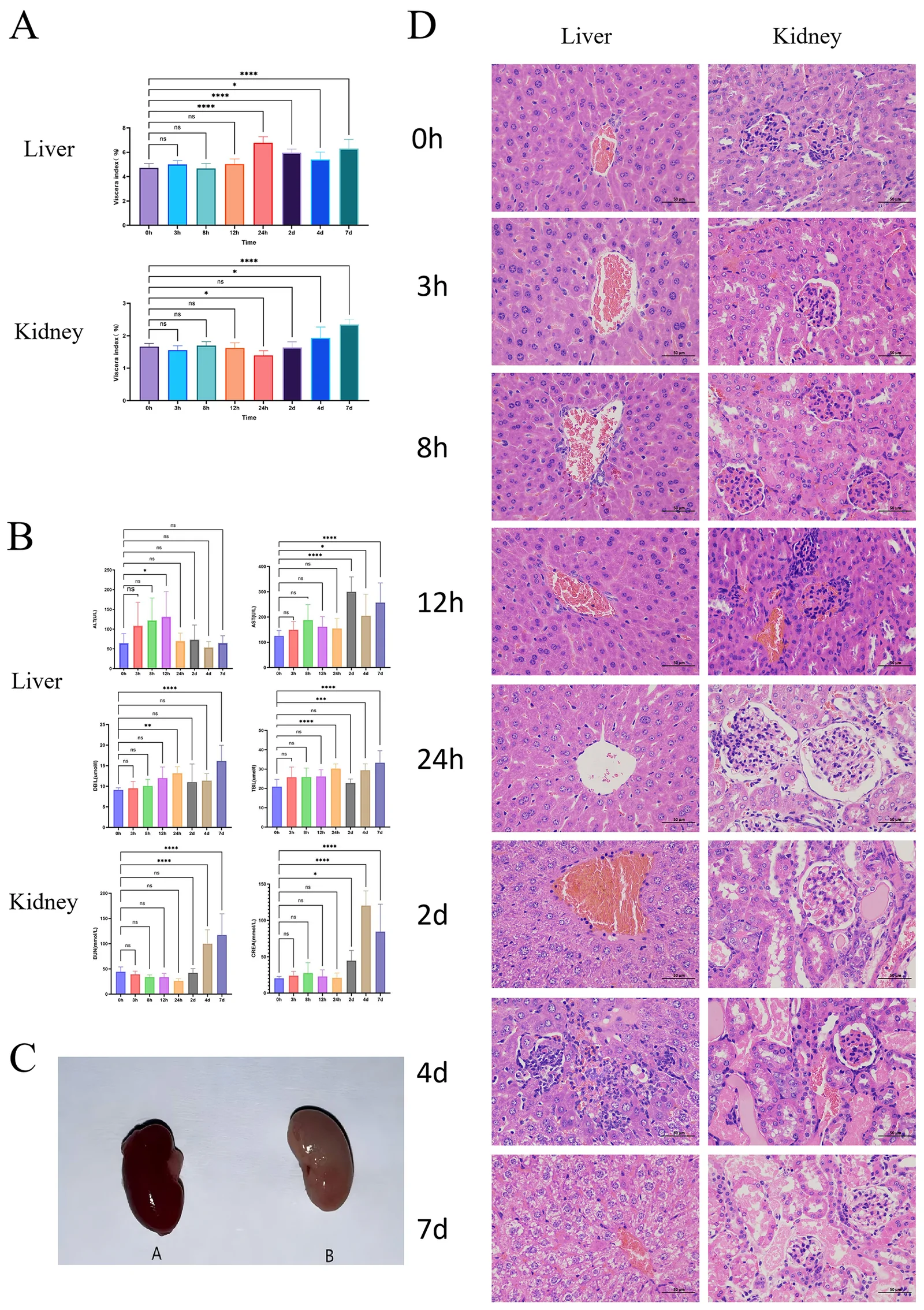
Indicators of α-amanitin-induced liver and kidney injury. (**A**) Organ indices of the liver and kidney. (**B**) Biochemical indicators of the liver and kidney. The significance levels are represented as follows: ns: *p* > 0.05, * *p* < 0.05, ** *p* < 0.01, *** *p* < 0.001, **** *p* < 0.0001. (**C**) Macroscopic observation of the kidneys two days after administration (A: control groups; B: treatment groups). (**D**) H&E staining of the liver and kidney at different time points.

The liver and kidney indices for the experimental groups at different time points relative to the 0 h group are presented in Table 1 and Figure 2A. Over time, both liver and kidney indices exhibited a general upward trend, and the statistical differences became increasingly pronounced with extended treatment duration, as illustrated in Figure 2A.

#### 3.3.2. Biochemical Index Assay

The biochemical indices of liver and kidney function for the experimental groups at different time points relative to the 0 h group are presented in Figure 2B. The α-amanitin induced both hepatic and renal dysfunction in mice. Among the liver function markers, ALT levels were the first to increase, beginning at 12 h. AST levels showed highly significant elevations from 2 d to 7 d, while DBIL and TBIL levels gradually increased from 24 h to 7 d. Regarding kidney function markers, BUN and CREA levels were significantly elevated from 2 d to 7 d (Figure 2B).

#### 3.3.3. Histopathological Analysis

Macroscopic examination revealed no significant changes in liver appearance compared with the control group. Conversely, the kidneys exhibited noticeable pallor starting from the 2d treatment group (Figure 2C).

Histopathological findings in the liver and kidney for the experimental groups at different time points relative to the 0 h group are summarized in Figure 2D. No significant lesions were observed in the liver or kidney tissues in the 3 h and 8 h groups. With prolonged exposure, focal vacuolar degeneration developed in the liver. Beginning at 24 h group, the liver exhibited disrupted tissue architecture and scattered inflammatory cell infiltration. Renal histopathology revealed focal inflammatory cell infiltration, dilation of Bowman’s capsule, thinning of the renal tubular walls, enlargement of the tubular lumina and the presence of cellular debris within the lumina. Beginning with the 2d group, casts were observed within Bowman’s capsules. In the 4 d group, renal tubules exhibited necrosis, nuclear loss, cell lysis and homogeneous eosinophilic staining (Figure 2D).

#### 3.3.4. Analysis of Differential Metabolites at Different Time Points

**Figure 3:**
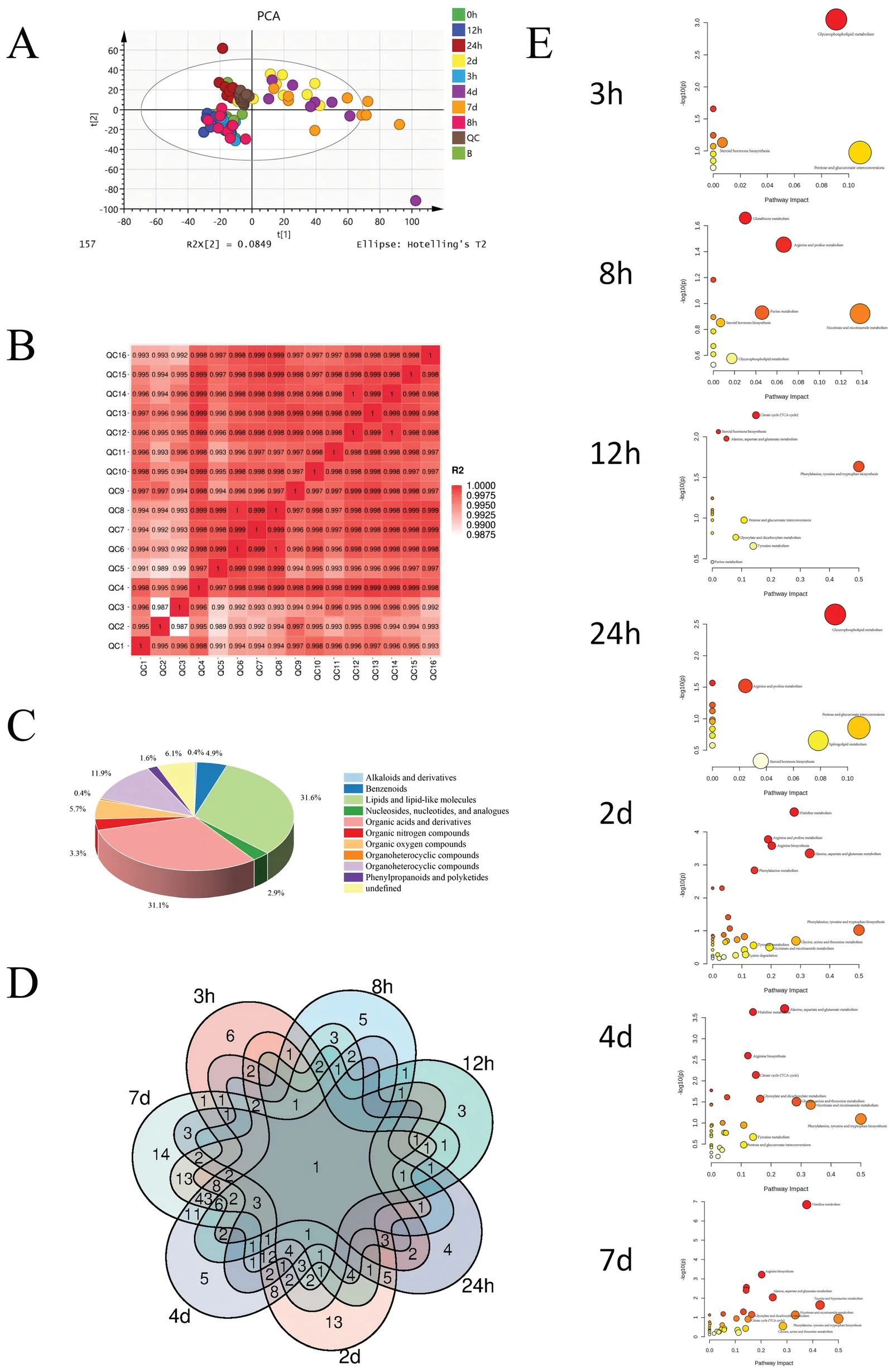
Untargeted metabolomics analysis. (**A**) PCA. (**B**) Correlation map of QC. (**C**) Statistical classification of differential metabolites by chemical category. (**D**) Venn diagram of differential metabolites at different time points. (**E**) Bubble charts of metabolic pathways at different time points.

PCA was performed on the peaks extracted from the experimental and QC samples. In both positive and negative ion modes, QC samples clustered tightly and Pearson correlation analysis yielded coefficients greater than 0.9, which confirmed excellent experimental reproducibility (Figure 3A,B). OPLS-DA was subsequently applied to differentiate the control and experimental groups at each time point and to identify differential metabolites. Model validity was confirmed via 200-permutation tests; all groups with the exception of the 0 h group demonstrated no overfitting, with Q^2^ regression lines intercepting below zero, thereby validating the reliability of the model.

Differential metabolites were identified based on the following criteria: *p* < 0.05, VIP ≥ 1.0 and |log_2_FC| ≥ 0.585. Compounds were preliminarily identified by matching features against the Human Metabolome Database (HMDB), PubChem and the Kyoto Encyclopedia of Genes and Genomes (KEGG). In both ion modes, 244 metabolites were identified in plasma samples. Based on the HMDB superclass system, the results revealed that lipids and lipid-like molecules (77 metabolites, 31.6%) and organic acids and derivatives (76 metabolites, 31.1%) were the predominant classes (Figure 3C).

Compared with the control group, the numbers of differential metabolites identified at 3 h, 8 h, 12 h, 24 h, 2 d, 4 d, and 7 d were 46, 41, 39, 56, 164, 148, and 170, respectively. Propionylcarnitine was the only differential metabolite identified across all time points (Figure 3D).

#### 3.3.5. Analysis of Metabolic Pathways at Different Time Points

Metabolic pathways analysis of the identified differential metabolites was performed using the MetaboAnalyst database. Pathways with impact > 0 were selected for further analysis, and a corresponding bubble plot was generated in Figure 3E.

### 3.4. Integrated Analysis of Network Toxicology and Metabolomics

#### 3.4.1. Compound–Reaction–Enzyme–Gene Networks

Using the MetScape plugin in Cytoscape, compound–reaction–enzyme–gene networks were constructed for each time point to identify the potential candidate targets and metabolic pathways implicated in α-amanitin-induced hepatorenal injury. These results were integrated with the potential candidate targets previously identified via network toxicology (IFNG, PTGS2, TP53, PPARG, GAPDH, C3, F2, and CYCS) for comprehensive analysis (Figure S3). Furthermore, compound–reaction–enzyme–gene networks were constructed based on differential metabolites identified by metabolomics. Integration of these networks with target genes from the network toxicology analysis revealed that PTGS2 was significantly enriched at the 3 h, 12 h, 2d, and 7 d time points.

#### 3.4.2. Molecular Docking

**Figure 4:**
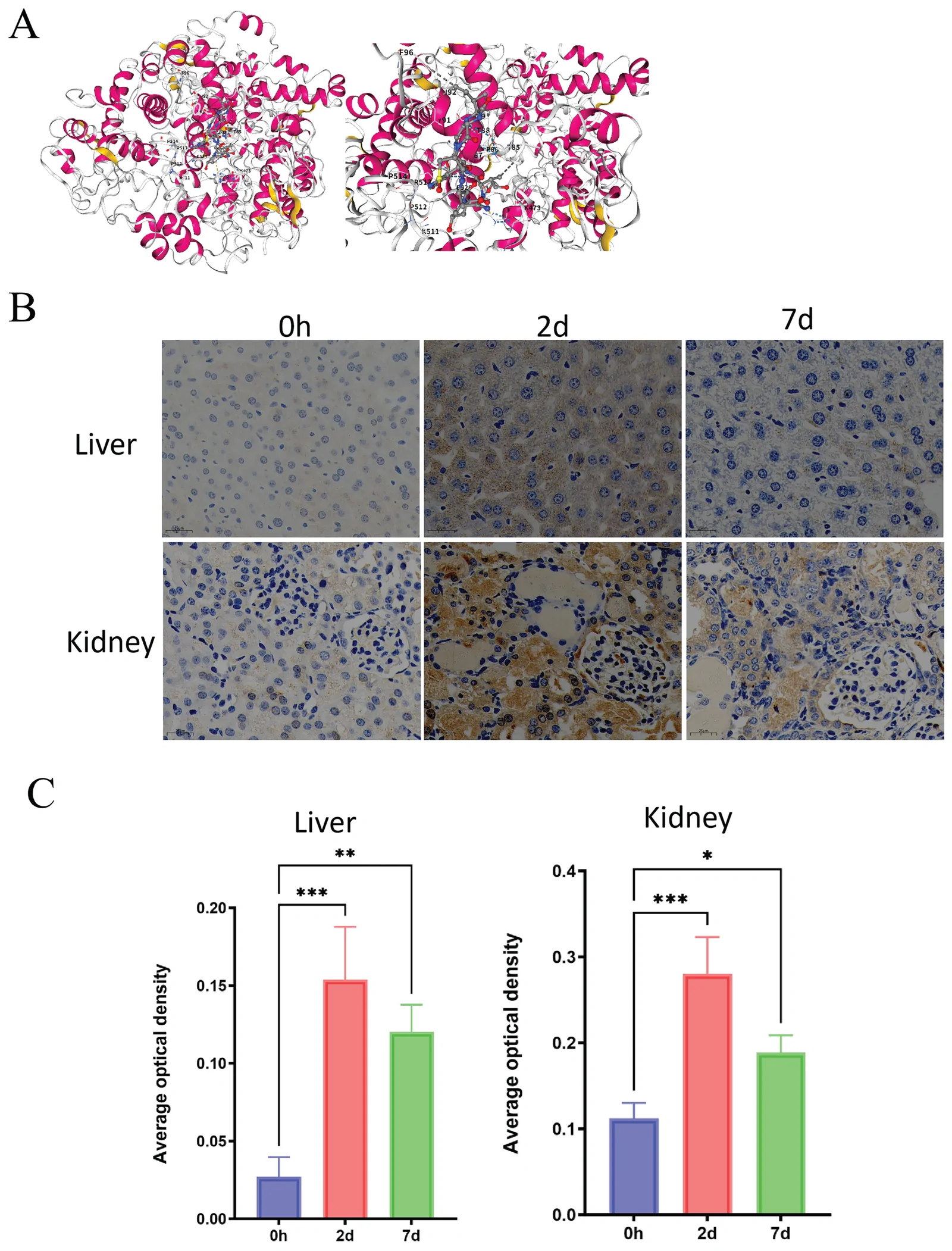
Integrated analysis of metabolomics and network toxicology. (**A**) Molecular docking result of α-amanitin and PTGS2. (**B**) Immunohistochemical results of PTGS2 in liver and kidney tissues at different time points. (**C**) AOD of PTGS2 in liver and kidney tissues at different time points. The significance levels are represented as follows: * *p* < 0.05, ** *p* < 0.01 and *** *p* < 0.001.

Molecular docking was performed to evaluate the binding affinity of α-amanitin to the identified potential candidate target. The Vina score served as a proxy for binding affinity, with more negative values indicating stronger receptor–ligand interactions (Figure 4A). A threshold of −5.0 kcal/mol was generally considered indicative of biological activity, whereas scores above this value required further experimental validation. A score below −8.0 kcal/mol suggested strong binding activity between the compound and the core target protein. In this study, the Vina score for α-amanitin binding to PTGS2 was −10.8 kcal/mol, indicating favorable and stable binding activity.

#### 3.4.3. Immunohistochemical Analysis

Because the detection window for α-amanitin in blood was within 48 h, two critical time points, 2 d and 7 d (with a longer detection period), were preliminarily selected for immunohistochemical validation. Compared with the 0 h group, the average optical density (AOD) values at both 2 d and 7 d showed statistically significant differences, exhibiting an overall trend of an initial increase followed by decrease in immunohistochemical analysis. Consistently, PTGS2 staining intensity also showed an initial enhancement followed by a subsequent reduction (Figure 4B,C). Additional time points will be considered in subsequent research.

## 4. Discussion

The genus *Amanita* is a large, globally distributed group within the order *Agaricales*, comprising approximately 600 described species [30]. Their striking morphological resemblance to edible mushrooms frequently leads to accidental ingestion. In 2024 alone, 599 mushroom poisoning incidents involving 1486 individuals were reported in China [31]. Among the amatoxins, α-amanitin is recognized as the most potent [32]. Thus, elucidating the mechanisms and biomarkers of α-amanitin-induced hepatorenal injury is crucial for understanding the broader toxicological profile of *Amanita* species. This study integrated network toxicology, untargeted metabolomics, molecular docking and histological approaches to preliminarily identify potential candidate targets, differential metabolites, and metabolic pathways involved in α-amanitin-induced hepatorenal injury, thereby providing novel insights into the systemic investigation of this potent toxin.

Previous network toxicology studies on α-amanitin have primarily focused on its hepatotoxicity effects [33]. However, as α-amanitin affects both the liver and kidneys, focusing solely on a single organ is insufficient to fully elucidate its toxic mechanisms [34,35]. Consequently, this study utilized a combined hepatic and renal toxicity prediction model to characterize the core mechanisms underlying α-amanitin-induced hepatorenal injury. The metabolic pathways involved in α-amanitin-induced hepatorenal injury, were obtained through network toxicology. Among these pathways, the complement and coagulation cascades pathway plays a critical role in maintaining immune defense and hemostatic homeostasis in humans [36]. Under normal physiological conditions, the complement system facilitates the clearance of pathogens and damaged cells, while the coagulation system regulates hemostasis. However, hepatic and renal injuries resulting from ischemia–reperfusion, inflammation and chemical poisoning frequently lead to the overactivation of these systems [37]. The combined action of complement and thrombin accelerates thrombosis and amplifies inflammation, ultimately resulting in organ failure [38]. These findings provide a theoretical foundation for the subsequent metabolomics analysis of plasma.

In this untargeted metabolomics study, all metabolite annotations were based on MS/MS spectral matching against public databases without authentic standard confirmation. Therefore, the identified metabolites were considered as tentatively annotated candidates rather than definitively confirmed compounds. Plasma metabolomic analysis of mice poisoned with α-amanitin preliminarily indicated that propionylcarnitine was the common differential metabolite across all seven time points. This finding is consistent with a reported metabolomics study in which 33 patients with amatoxin poisoning were classified into survival and death groups [39]. Propionylcarnitine might serve as a potential poisoning marker for α-amanitin-induced hepatorenal injury, providing guidance for poisoning treatment and identification in mushroom poisoning events caused by *Amanita* species. Propionylcarnitine is an important endogenous metabolic intermediate formed by the conjugation of L-carnitine with propionyl-CoA [40], and it plays a critical role in the branched-chain amino acid metabolism pathway and fatty acid *β*-oxidation pathway. Under normal physiological conditions, propionylcarnitine concentrations are very low. However, when metabolic disorders, such as propionemia or mitochondrial enzyme defects occur, propionylcarnitine accumulates extensively in the blood and could be widely used as an early screening marker [41,42,43]. In our study, propionylcarnitine was upregulated at the studied time points following α-amanitin administration compared to the control group, indicating that α-amanitin caused profound systemic metabolic disturbances.

Plasma metabolomic analysis of mice poisoned with α-amanitin preliminarily revealed that plasma metabolites exhibited a temporally dynamic progression across the 3 h, 8 h, 12 h, 24 h, 2 d, 4 d and 7 d groups, which was consistent with the toxicological injury–response–adaptation model. At 3 h, the pentose and glucuronate interconversions pathway emerged as a critical detoxification mechanism, facilitating the conversion of lipophilic compounds into water-soluble metabolites for excretion [44]. Glycerophospholipids, as major components of cell membranes, maintain membrane structural integrity and regulate various cellular functions [45]. Between 8 h and 12 h, in addition to the alterations in the aforementioned metabolic pathways, changes in redox homeostasis and core energy metabolism pathways were also observed. Glutathione metabolism was activated, nicotinate/nicotinamide metabolism was altered, and amino acid metabolism pathways (alanine, aspartate and glutamate) began to mediate TCA cycle activity and enhance gluconeogenesis. The period from 24 to 48 h was characterized by systemic metabolic dysregulation due to persistent stimulation. At 24 h, the sphingolipid metabolism pathway was enriched, with ceramide accumulation and reduced sphingosine-1-phosphate (S1P) promoting apoptosis and inflammatory responses [46,47]. At 48 h, the metabolic disturbances demonstrated multi-system synergy. Extensive abnormalities were observed in amino acid metabolism networks involving phenylalanine, tyrosine, tryptophan, arginine and proline, indicating inhibition of hepatic hydroxylase activity, urea cycle disorders and anaplerotic compensation due to mitochondrial dysfunction [48,49]. Concurrently, transamination and heme synthesis dependent on pyridoxal phosphate (vitamin B6) were impaired, along with specific alterations in primary bile acid and porphyrin metabolism, reflecting comprehensive damage to hepatobiliary excretion, detoxification capacity and coenzyme metabolism [50]. The period from 4 d to 7 d marked the phase of adaptive repair and functional reconstruction. One-carbon metabolism was activated during the late injury phase, providing nucleotide precursors for hepatorenal regeneration and supporting glutathione synthesis [51]. Although the TCA cycle, glycerophospholipid metabolism, and glutathione metabolism remained persistently active, the organism initiated alternative energy pathways, including a shift toward anaerobic pyruvate metabolism and activation of galactose metabolism. At 7 d, alterations in taurine and hypotaurine metabolism aimed to restore metabolic homeostasis in liver and kidney tissues, inhibiting the progression toward chronic injury through antioxidant effects, promotion of bile acid excretion, and membrane stabilization [52,53]. This temporally dynamic progression correlated with the clinical symptoms observed at different time points following poisoning.

PTGS2 was tentatively annotated as a potential candidate target involved in α-amanitin-induced hepatorenal injury by integrated analysis of the network toxicology and metabolomics datasets. 2-AG was tentatively annotated as its associated metabolite and the most abundant endocannabinoid in the human body; it serves as a major agonist of cannabinoid CB1 and CB2 receptors, exhibiting neuroprotective and anti-inflammatory effects [54]. The literature indicates that PTGS2 expression is upregulated during inflammatory pathological processes [55]. PTGS2 catalyzes the conversion of 2-AG into prostaglandin glycerol esters (PG-Gs) via a non-canonical metabolic pathway, thereby serving as a crucial link between arachidonic acid metabolism and the endocannabinoid system. The oxidation of 2-AG by PTGS2 resulted in its inactivation and decreased binding affinity for CB1 and CB2 receptors, representing a primary degradation pathway for 2-AG under inflammatory conditions. In our study, molecular docking confirmed a highly favorable and stable binding energy between α-amanitin and PTGS2. Immunohistochemistry analysis further demonstrated that PTGS2 expression was enhanced at two time points compared with 0 h, which was consistent with the increased infiltration of inflammatory cells observed in histopathological sections. PTGS2 protein expression showed an increase followed by a subsequent decrease, which may be attributed to the dynamic evolution of α-amanitin-induced injury. This trend aligned with the results of metabolomic pathway analysis, which indicated that the organism was in a state of metabolic dysregulation at 2 d and undergoing adaptive repair and functional reconstruction at 7 d. In conclusion, these results preliminarily revealed that PTGS2 may serve as a tentatively annotated target in α-amanitin-induced hepatorenal injury.

Current metabolomics research increasingly emphasizes the cautious interpretation of findings; the metabolites identified solely through data-driven pathway summarization remain unverified and should not be designated as biomarkers. This principle provides a reference for accurately characterizing key metabolites and offers guidance for subsequent data interpretation in metabolomics studies [56]. Consequently, the metabolomic findings of this study should be understood as exploratory results. We acknowledge that metabolite identifications in this untargeted metabolomics study are tentative and have not been confirmed by authentic standards or targeted LC-MS/MS, which represents a significant limitation. In particular, the key metabolites propionylcarnitine and 2-AG should be prioritized for validation in future studies using stable isotope-labeled internal standards and multiple reaction monitoring (MRM) methods. Although our computational and expression data strongly implicated PTGS2 in the cellular response to α-amanitin, these findings remain preliminary and correlational. In future studies, PTGS2 enzymatic activity assays, gene silencing and pharmacological inhibition should be strictly employed to validate whether PTGS2 directly mediates α-amanitin-induced toxicity. Nevertheless, pathway-level conclusions were derived from groups of consistently altered metabolites across multiple time points, providing collective biological relevance that was less dependent on the absolute confidence of any single compound identification.

This study has several limitations. The intraperitoneal route of α-amanitin administration did not replicate the oral route of actual poisoning incidents. The exclusive use of a single sex (male) and strain (KM mice) overlooks potential sex- and species-related differences. In addition, high-quality human clinical trials to validate the animal-based findings are absent. Future investigations should carefully consider the consistency between the administration route and actual poisoning exposure and incorporate both male and female animals, including validation in large animal models and human sample studies.

## 5. Conclusions

In this study, significant metabolic disturbances were induced in KM mice following intraperitoneal administration of α-amanitin at a dose of 0.2 mg/kg. Metabolomic analysis preliminarily revealed that α-amanitin-induced hepatorenal injury followed a temporally dynamic progression, which was consistent with the toxicological injury–response–adaptation model. By integrating network toxicology and untargeted metabolomics, PTGS2 was tentatively annotated as a potential candidate target, with propionylcarnitine and 2-AG as key associated metabolites. This study systematically investigated the latent mechanisms and markers of α-amanitin-induced hepatorenal injury in mice through the integration of network toxicology and metabolomics. Overall, these findings provide a reference for poisoning treatment and identification in mushroom poisoning events caused by *Amanita* species, while also enriching the research framework for understanding the toxicological mechanisms of natural fungal toxins.

## Figures and Tables

**Table 1:** Changes in organ indices (mean ± SD, *n* = 9).

Time	Organ Indices	
	Liver	Kidney
0 h	4.17 ± 0.36	1.67 ± 0.10
3 h	5.00 ± 0.32	1.55 ± 0.14
8 h	4.67 ± 0.41	1.70 ± 0.12
12 h	5.03 ± 0.44	1.62 ± 0.16
24 h	6.78 ± 0.51 ****	1.39 ± 0.14 *
2 d	5.96 ± 0.31 ****	1.63 ± 0.18
4 d	5.41 ± 0.60 *	1.93 ± 0.34 *
7 d	6.31 ± 0.75 ****	2.35 ± 0.16 ****

Notes: The significance levels are represented as follows: * *p* < 0.05, **** *p* < 0.0001.

## Data Availability

The original contributions presented in this study are included in the article/Supplementary Materials/https://ngdc.cncb.ac.cn/omix/view/OMIX018841 (accessed on 27 July 2026). Further inquiries can be directed to the corresponding author.

## Supplementary Materials

The following supporting information can be downloaded at https://www.mdpi.com/article/10.3390/metabo16080551/s1. Figure S1: Volcano plot of variation fold analysis in positive and negative ion modes. Figure S2: OPLS-DA score plots at different time points and the permutation test. Figure S3: Network diagram of compounds, reactions, enzymes and genes at different time points (3 h, 8 h, 12 h, 24 h, 2 d, 4 d and 7 d). The original figure 1, figure 2, figure 3 and figure 4 have been provided.

## Abbreviations

The following abbreviations are used in this manuscript:

PPI	Protein–protein interaction
QC	Quality control
BP	Biological process
CC	Cellular component
MF	Molecule function
VIP	Variable importance in projection
FC	Fold change
AOD	Average optical density
2-AG	2-arachidonoylglycerol
